# Vacuum-assisted biopsy in the era of low-risk ductal carcinoma *in situ* active monitoring: real world data and implications

**DOI:** 10.3389/fonc.2025.1618476

**Published:** 2025-10-24

**Authors:** Henrique Lima Couto, Carolina Nazareth Valadares, Aleida Nazareth Soares, Bernardo Ferreira de Paula Ricardo, Paola Hartung Toppa, Bertha Andrade Coelho, Eduardo Carvalho Pessoa, Vivian Resende, Tereza Cristina de Oliveira Ferreira, Andre Mattar, Andressa Amorim, Paula Clarke, Rufo Freitas-Junior, Gabriel de Almeida Silva Junior, Nisha Sharma, Henrique Silva Bartels, Charles Andreé Joseph de Paula, Geraldo Felício Cunha Júnior, Dênia Reis de Paula Oliveira, Bruna Torres Silvestre da Silva, Daniela Rodrigues Siqueira, Marcus Simões Castilho, Marcelo Antonini, Heverton Leal Ernesto de Amorim, Jane Sanglard de Oliveira, Bruna Antunes de Miranda Pires, Douglas de Miranda Pires, Shirley das Graças Ferreira, Thais Paiva Moraes, Larissa Barbosa Oliveira, Paula Martins Cristina Soares, Vilmar Marques de Oliveira, Annamaria Massaud Rodrigues dos Santos, Rosemar Macedo Souza Rahal, Augusto Tufi Hassan, Clécio Ênio Murta de Lucena, Giuliano Tosello, Daniel de Araújo Brito Buttros, Guilherme Novita, Romana Giordana Ribeiro Saliba, Bárbara Pace Silva Assis Carvalho, Waldeir José de Almeida Junior, Marcellus do Nascimento Moreira Ramos, Ana Carolina Guglielmelli Mendonça, Fernando Marcos Reis

**Affiliations:** 1 Redimama-Redimasto Breast Unit, Belo Horizonte, Minas Gerais, Brazil; 2 Brazilian Society of Mastology, Belo Horizonte, Minas Gerais, Brazil; 3 Hospital Paulistano, São Paulo, São Paulo, Brazil; 4 Brazilian Society of Mastology, Rio de Janeiro, Rio de Janeiro, Brazil; 5 Faculdade de Saúde Santa Casa BH, Belo Horizonte, Minas Gerais, Brazil; 6 Faculty of Medical Sciences of Minas Gerais, Belo Horizonte, Minas Gerais, Brazil; 7 Anatomia Laboratory, Belo Horizonte, Minas Gerais, Brazil; 8 Mater Clínica, Montes Claros, Minas Gerais, Brazil; 9 UNIFIPMOC University Center, Montes Claros, Minas Gerais, Brazil; 10 São Paulo State University Júlio de Mesquita Filho, School of Medicine, Botucatu, Brazil; 11 Brazilian Society of Mastology, São Paulo, São Paulo, Brazil; 12 Faculty of Medicine, Federal University of Minas Gerais, Belo Horizonte, Minas Gerais, Brazil; 13 Women Hospital, São Paulo, São Paulo, Brazil; 14 Military Hospital of the Institute of Social Security of Military Servants of Minas Gerais, Belo Horizonte, Brazil; 15 Federal University of Goiás, Goiânia, Goiás, Brazil; 16 Orizonti Institute, Belo Horizonte, Minas Gerais, Brazil; 17 Leeds Teaching Hospitals NHS Trust, Leeds, United Kingdom; 18 CETUS Oncology, Belo Horizonte, Minas Gerais, Brazil; 19 Radiocare, Belo Horizonte, Minas Gerais, Brazil; 20 Hospital of the State Public Servant of São Paulo, São Paulo, Brazil; 21 UD Diagnóstica, João Pessoa, Paraíba, Brazil; 22 Santa Casa de Misericórdia Hospital, Belo Horizonte, Minas Gerais, Brazil; 23 Mater Dei Hospital, Belo Horizonte, Minas Gerais, Brazil; 24 Faculty of Medical Sciences, Santa Casa of Sao Paulo, São Paulo, São Paulo, Brazil; 25 Institute of Social Security for State Employees of Minas Gerais (IPSEMG), Belo Horizonte, Minas Gerais, Brazil; 26 Oncoclínicas - CAM, Salvador, Bahia, Brazil; 27 Western Paulista Cancer Institute, Presidente Prudente, São Paulo, Brazil; 28 Faculty of Medicine of Claretiano University, Rio Claro, Brazil; 29 Oncoclinicas Group, São Paulo, São Paulo, Brazil; 30 Sonar Breast Unit, Belo Horizonte, Minas Gerais, Brazil

**Keywords:** breast cancer, vacuum assisted biopsy, enlarged vacuum assisted biopsy, vacuum assisted excision, DCIS, active monitoring

## Abstract

**Background:**

The short-term oncological safe of active monitoring for ductal carcinoma *in situ* (DCIS) with low risk (LR-DCIS) of progression to invasive cancers (IC) has been demonstrated. This study evaluates vacuum assisted biopsy (VAB) as diagnostic test for LR-DCIS active monitoring (AM) in real-world clinical practice.

**Methods:**

Database analysis of 116 cancers [both invasive breast cancers (IC) and ductal carcinoma *in situ* (DCIS)] diagnosed by VAB submitted to standard surgical treatment with complete histological data from VAB and surgery from 04/13/2017 to 11/28/2020. The VAB results matched the surgical pathology, considered the gold standard, and AM criteria. The pathological diagnoses were grouped into malignancies requiring guideline surgical treatment [DCIS with high risk (HR-DCIS) of progression to IC or IC] versus those eligible to alternative AM (LR-DCIS). HR-DCIS/IC were considered positive while LR-DCIS negative results. VAB sensitivity, specificity, positive predictive value (PPV), negative predictive value (NPV), and accuracy were obtained.

**Results:**

Mean age 55.6 [± 12.27]; mean IC size 7.14 [± 5.17]mm and 12.6 [± 11.63]mm for DCIS. Out of 116 malignancies diagnosed by VAB, 15 (12.9%) resulted LR-DCIS in the biopsy, 10 (8.6%) confirmed LR-DCIS in surgery, and 5 (4.3%) upgraded to HR-DCIS/IC in surgery. VAB showed 95.28% (89.3–98.5; 95% CI) sensitivity, 100% (69.2–100; 95% CI) specificity, PPV was 100% (96.4–100; 95% CI), and NPV 66.67% (38.4–88.2; 95% CI). VAB LR-DCIS AM was 6.9% (8/116) and underdiagnoses 2.6% (2 pT1a-bN0 hormone receptor positive and 1 HR-DCIS).

**Conclusion:**

VAB LR-DCIS AM would lead to a moderate (6.9%) overall reduction of short-term breast cancer surgical overtreatment counterbalanced by a low rate (2.6%) of underdiagnosed HR-DCIS/IC potentially treatable by adjuvant hormone therapy.

**Clinical Trial Registration:**

https://plataformabrasil.saude.gov.br/visao/pesquisador/gerirPesquisa/gerirPesquisaAgrupador.jsf, identifier 25761019.8.0000.5138.

## Highlights

- VAB is excellent in selecting breast cancer patients to guideline surgical treatment.
- VAB LR-DCIS active monitoring reduces breast cancer surgical overtreatment by 6.9%.
- IC overall undertreatment of VAB LR-DCIS active monitoring is 1.7%.
- Enlarged VAB is not superior to ordinary VAB in diagnosing LR-DCIS.

## 1 Introduction and objectives

The management of breast cancer has transitioned from generalized, radical treatments, such as radical mastectomy for all, to personalized and de-escalated strategies, incorporating targeted therapies and breast-conserving surgery (1). Similarly, breast cancer diagnosis has evolved from diagnostic surgery and incisional biopsies to minimally invasive percutaneous procedures, including fine-needle aspiration (FNA), tru-cut core needle biopsies (CNB), and vacuum-assisted biopsy (VAB) (2).

Accurate histological diagnosis is essential for optimal therapeutic planning, aiming to achieve effective disease control while minimizing aesthetic and functional sequelae (3). Historically, cytological diagnosis via FNA was sufficient to initiate surgical treatment; however, in contemporary practice, precise histological and immunohistochemical diagnosis has become indispensable (2). In the context of personalized medicine, accurate percutaneous diagnosis is crucial for identifying breast malignancies that require immediate surgical, systemic, or radiotherapeutic interventions (4).

While distinguishing ductal carcinoma *in situ* (DCIS) from invasive carcinomas (IC) was previously a primary objective, the advent of de-escalated therapeutic approaches necessitates more nuanced diagnostic stratification (2). Specifically, differentiating DCIS with low risk (LR-DCIS) of progression to IC, with its favorable prognosis and potential for active monitoring (AM), from DCIS with high risk (HR-DCIS) of progression to IC is of paramount importance (5–17).

This study objective is to evaluate the diagnostic performance and clinical implications of VAB for LR-DCIS in real-world practice, within the framework of personalized medicine and emerging de-escalation strategies (5–19). The security for AM of LR-DCIS is dependent on the underdiagnosis risk of the method used. In this study, we compared results of VAB with the final surgical pathology and evaluated its impact in real world practice according to the eligibility criteria established in the COMET trial (18).

## 2 Methods

### 2.1 Patient eligibility and study design

The study was approved by the Ethics Committee of Santa Casa of Belo Horizonte under the number 25761019.8.0000.5138, and all methods were conducted in accordance with national guidelines. Written informed consent was obtained from all participants. The data set used and analyzed during the study is available upon reasonable request to the corresponding author.

**Figure 1:**
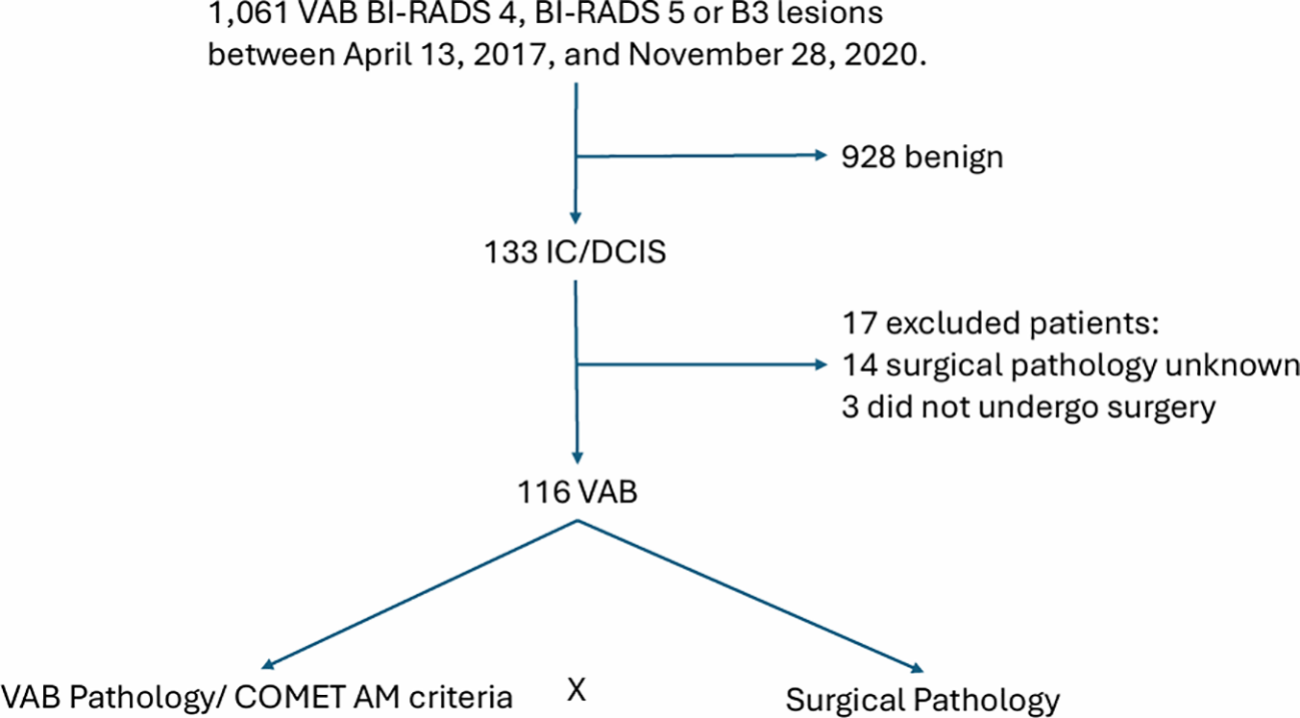
Study design.

A total of 1,061 vacuum-assisted biopsies (VAB) for suspicious breast lesions classified as BI-RADS 4, BI-RADS 5, or lesions with indeterminate malignant potential from prior CNB (B3 lesions per The Royal College of Pathologists) were performed at a dedicated breast diagnostic unit in Brazil between April 13, 2017, and November 28, 2020. Patients with benign histology on VAB, confirmed malignancy without primary surgical treatment, or unavailable final surgical pathology were excluded. The final study population included 116 women diagnosed with IC and DCIS with complete VAB and surgical pathology reports which were included in the analysis (**Figure 1**).

Baseline demographic data was recorded. Imaging data were collected, including baseline assessments, findings (mass ± calcification), the image-guided approach used for VAB (ultrasound or stereotactic), and the maximum imaging tumor size (TI).

### 2.2 VAB procedure

A diagnostic VAB was performed. Following each procedure, a mammogram was obtained to confirm the position of the clip marker. VAB were classified as either ordinary VAB (OVAB) or enlarged VAB (EVAB). OVAB is defined by taking less than12 core samples with a 7G needle or 18 core samples with a 10G needle, eventually the lesion is completely excised. EVAB was defined by complete lesion excision as confirmed by imaging or retrieval of more than 12 core samples with a 7G needle or 18 core samples with a 10G needle (20). The choice of biopsy device (EnCor Enspire™ Breast Biopsy System – BD or Mammotome Revolve™ Dual Vacuum Assisted Breast Biopsy System) and needle gauge was at discretion of the operating physician.

### 2.3 VAB/surgical pathological reports

Gross specimens were separated from clots, measured, weighed, and inked. All fragments were entirely included, and slices were sectioned at four-micron thickness. Cases typically ranged from one to five paraffin blocks. Histological evaluation included standard hematoxylin-eosin (HE) staining, with additional immunohistochemistry performed at the pathologist’s discretion. Fluorescence *in situ* hybridization (FISH) and genetic analyses, such as Oncotype, were conducted if indicated.

All tissue samples underwent comprehensive histopathological evaluation. Pathological assessment included measurement of the maximum tumor size, determination of diagnosis (IC ± DCIS), presence of DCIS with necrosis, multifocality (surgical specimen), biomarker status (ER, PR, HER2, Ki67), morphological tumor type, and nuclear and histological grades. The maximum pathological tumor size following VAB was defined as the largest tumor dimension observed on the slide containing the most extensive tumor involvement (21). Sentinel node biopsy (SNB) was performed according to standard clinical practice (22). The presence of residual invasive or *in situ* disease in the surgical specimen was documented. The pathological reports followed The College of American Pathologists Guidelines and World Health Organization Classification of Tumors of the Breast (23–31).

HR-DCIS was defined as any high-grade ductal carcinoma *in situ*, while LR-DCIS was defined as low- or intermediate-grade ductal carcinoma *in situ* with or without necrosis (7, 18).

For multicentric or bilateral breast cancers, only the tumor measurements and outcomes related to the lesion sampled by VAB were analyzed. One patient with two multicentric nodules underwent separate VAB procedures for each lesion; these were treated as distinct cases. These cases were automatically excluded for potential AM.

All cases underwent surgical excision following VAB. Postoperatively, radiography of the surgical specimen was performed to confirm the presence of the marker placed during VAB.

### 2.4 Diagnostic test statistical evaluation

An exploratory analysis was initially conducted to assess the normality of the data with continuous distribution. To this end, the Shapiro-Wilk test was used. For continuous variables, measures of central tendency (mean and median) and dispersion (standard deviation) were obtained. For categorical variables, frequency and percentage for each category were calculated.

OVAB and EVAB variables were compared to evaluate potential selection bias and disparities in the cohort that could influence the results. For continuous variables (OVAB vs. EVAB), comparisons were performed using the Mann-Whitney test, which is applied in pairwise comparisons of unpaired samples. Fisher’s Exact Test was used for comparisons between frequencies obtained in each categorical variable. This test was chosen due to the characteristics of the analyzed sample and the presence of very low values, which made it impossible to apply the Chi-square test across all variables. Therefore, to ensure consistency in the analysis, Fisher’s Exact Test was adopted for all frequency comparisons. In all analyses performed, the obtained differences were considered statistically significant when the p-value was less than or equal to 0.05 (p ≤ 0.05).

To evaluate the diagnostic test, the pathological results of OVAB and EVAB were analyzed both separately and collectively (VAB), using surgical pathology as the gold standard for comparison. Pathological diagnoses were categorized into malignancies requiring guideline surgical treatment versus those eligible for potential AM. Lesions necessitating guideline surgical intervention were classified as positive and included IC and HR-DCIS. Lesions eligible for AM were classified as negative and included LR-DCIS.

To evaluate the association between VAB results and the surgical gold standard, 2x2 contingency tables were analyzed. VAB HR-DCIS/IC (positive) and VAB LR-DCIS (negative) were compared to surgical final pathology. Diagnostic performance metrics, including sensitivity, specificity, positive predictive value (PPV), negative predictive value (NPV), and accuracy, were calculated for each comparison with a confident interval (CI) of 95%. VAB LR-DCIS results were matched to COMET inclusion and exclusion criteria (18) (**Table 1**).

**Table 1:** COMET inclusion/exclusion criteria (18).

COMET criteria	Inclusion	Exclusion
Age	≥40	<40
DCIS	Grade 1 or 2	Grade 3
HR	+	–
HER 2	–	+
Lesion type	Calcifications	mass
Symptoms	Absent	Present

DCIS, ductal carcinoma *in situ*; HR, hormone receptor; HER 2, Human Epidermal growth factor Receptor-type 2. Her 2-: 0-1/3+ in immunohistochemistry or 2/3+ in immunohistochemistry with fluorescence *in situ* hybridization (FISH) negative; HER 2+: 3/3+ in immunohistochemistry or 2/3+ in immunohistochemistry with fluorescence *in situ* hybridization (FISH) positive.

Statistical analyses were performed using Graphpad Prism^®^ software (GraphPad Software, version 8.0, La Jolla California USA, www.graphpad.com) for Windows, the GraphPad QuickCalcs software for detecting potential outlier values, and Stata^®^ (version 14.0, Stata Corporation, College Station, TX, USA).

Statistical analyses of the diagnostic test performance were conducted using Stata^®^ (version 14.0, Stata Corporation, College Station, TX, USA) employing the *diagt* command to estimate sensitivity, specificity, PPV, NPV, and their corresponding 95% confidence intervals.

## 3 Results

### 3.1 Cohort

In the general study population, the mean age was 55.66 years (± 12.27). The mean final tumor size was 7.14 mm (± 5.17) for IC (T) and 12.61 mm (± 11.63) for DCIS. Among the cases, 56.03% underwent EVAB, while 43.97% underwent OVAB (**Table 2**).

**Table 2:** Descriptive and comparative analysis of continuous variables (VAB, EVAB and OVAB).

Continuous variables	Mean	± SD	Median	P25 - P75	Min - max	*P* value ^MW^
Age (years)						0,0855
VAB (n=116)	55,66	± 12,27	56,00	46 - 65	20 - 91	
EVAB (n=65)	57,38	± 12,78	58,00	48 - 66	31 - 91	
OVAB (n=51)	53,47	± 11,35	51,00	45 - 63	20 - 76	
Largest image size (mm) (n=78)						0,0175^*^
VAB (n=78)	11,67	± 10,59	9,00	6,95 - 13,25	4 - 88	
EVAB (n=59)	9,58	± 4,64	9,00	6 - 10	5 - 26	
OVAB (n=19)	18,15	± 18,75	14,00	8 - 25	4 - 88	
Largest VAB T measurement (mm) (n=106)						0,0451^*^
VAB (n=106)	5,29	± 2,89	5,00	4 - 6,63	1 - 25	
EVAB (n=61)	5,81	± 3,35	5,00	4 - 7	1,75 - 25	
OVAB (n=45)	4,58	± 1,95	4,00	3,13 - 6	1 - 9	
Residual IC T (n=113)						0,2694
VAB (n=113)	2,95	± 5,48	0,00	0 - 3	0 - 25	
EVAB (n=64)	3,27	± 5,61	0,00	0 - 4	0 - 25	
OVAB (n=49)	2,53	± 5,34	0,00	0 - 1	0 - 23	
Residual DCIS T (n=110)						<0,0001^*^
VAB (n=110)	7,57	± 11,25	2,00	0 - 13,25	0 - 65	
EVAB (n=62)	3,58	± 6,72	0,00	0 - 3,25	0 - 30	
OVAB (n=48)	12,73	± 13,66	10,00	0 - 20,75	0 - 65	
ER (%) (n=114)						0,0001^*^
VAB (n=114)	68,75	± 39,34	90,00	40 - 100	0 - 100	
EVAB (n=65)	79,82	± 33,11	100,00	70 - 100	0 - 100	
OVAB (n=49)	54,08	± 42,39	70,00	0 - 95	0 - 100	
PR (%) (n=114)						<0,0001^*^
VAB (n=114)	50,44	± 41,48	60,00	0,75 - 90	0 - 100	
EVAB (n=65)	63,15	± 40,50	80,00	10 - 100	0 - 100	
OVAB (n=49)	33,57	± 36,80	10,00	0 - 80	0 - 100	
KI67 (%) (n=112)						0,0109^*^
VAB (n=112)	22,07	± 19,11	20,00	10 - 30	2 - 90	
EVAB (n=65)	18,94	± 18,04	10,00	5 - 25	2 - 80	
OVAB (n=47)	26,40	± 19,89	20,00	10 - 30	2 - 90	
Final IC T (mm) (n=76)						0,1702
VAB (n=76)	7,14	± 5,17	6,00	4 - 9,75	0,8 - 25	
EVAB (n=51)	7,45	± 4,68	6,00	4 - 9	2 - 25	
OVAB (n=25)	6,51	± 6,10	5,00	1 - 10	0,8 - 23	
Final DCIS T (mm) (n=37)						0,1673
VAB (n=37)	12,61	± 11,63	8,00	5 - 19	2 - 65	
EVAB (n=12)	8,29	± 5,22	7,00	4,25 - 11	2,5 - 20	
OVAB (n=25)	14,68	± 13,29	13,00	5 - 20,50	2 - 65	

VAB, vacuum assisted biopsy; OVAB, ordinary vacuum assisted biopsy; EVAB, enlarged vacuum assisted biopsy; SD, standard deviation; P25, 25th percentile; P75, 75th percentile; Min, minimum value; Max, maximum value. MW, Mann-Whitney test; * statistical significance (p ≤ 0.05).

Bold values: Statistically significant.

Patients undergoing EVAB demonstrated statistically higher median values compared with the OVAB group for the following parameters: largest VAB tumor size, estrogen receptor (ER) expression, and progesterone receptor (PR) expression. In contrast, OVAB patients exhibited statistically higher medians compared with EVAB patients for the following parameters: largest image size, residual DCIS tumor size, and Ki67 index (**Table 2**).

For categorical variables, the general study population demonstrated the following characteristics: 91.38% of procedures were performed by a single physician, 65.52% were ultrasound-guided, and 42.24% involved masses only. Unifocal lesions were present in 87.07% of cases, and 95.69% were not multicentric or bilateral. Intermediate nuclear grade was observed in 48.28% of cases. In the final pathology 67,24% were IC and 32.75% were DCIS. Among surgical interventions, 77.39% were lumpectomies (**Table 3**).

**Table 3:** Descriptive and comparative analysis of categorical variables (VAB, EVAB, and OVAB).

Categorical variables	VAB (n=116)		EVAB (n=65)		OVAB (n=51)		*P* value ^F^
	N	%	N	%	N	%	
Performing physician							
Dr. 1	106	91,38	59	90,77	47	92,16	0,932
Dr. 2	4	3,45	2	3,08	2	3,92	
Dr. 3	4	3,45	3	4,62	1	1,96	
Dr. 4	2	1,72	1	1,54	1	1,96	
US or MMG							
US	76	65,52	56	86,15	20	39,22	<0,0001^*^
MMG	40	34,48	9	13,85	31	60,78	
Mass/calcifications							
Mass	49	42,24	40	61,54	9	17,65	<0,0001^*^
Mass + Calcs	31	26,72	19	29,23	12	23,53	
Calcs	36	31,03	6	9,23	30	58,82	
Multifocal							
No	101	87,07	58	89,23	43	84,31	0,579
Yes	15	12,93	7	10,77	8	15,69	
Multicentric/bilateral							
No	111	95,69	64	98,46	47	92,16	0,167
Yes	5	4,31	1	1,54	4	7,84	
VAB pathology							
Invasive Cancer (IC)	26	22,41	22	33,85	4	7,84	<0,0001^*^
DCIS	43	37,07	15	23,08	28	54,90	
IC + DCIS	36	31,03	26	40,00	10	19,61	
DCIS + microinvasion	11	9,48	2	3,08	9	17,65	
DCIS with comedonecrosis							
No	59	50,86	45	69,23	14	27,45	<0,0001^*^
Yes	56	48,28	19	29,23	37	72,55	
DCIS absent	1	0,86	1	1,54	0	0,00	
Histological grade (n=68)							
Low	18	26,47	16	30,77	2	12,50	0,204
Intermediate	34	50,00	26	50,00	8	50,00	
High	16	23,53	10	19,23	6	37,50
Nuclear grade							
Low	12	10,34	9	13,85	3	5,88	0,031
Intermediate	56	48,28	36	55,38	20	39,22	
High	48	41,38	20	30,77	28	54,90	
Final pathology							
Invasive cancer	18	15,52	15	23,08	3	5,88	<0,0001*
Invasive cancer + DCIS	52	44,83	37	56,92	15	29,41	
DCIS	38	32,75	12	18,46	26	50,98	
CDIS + microinvasion	8	6,90	1	1,54	7	13,73	
Axillary nodes pathology (n=84)							
pN0	75	89,29	45	88,24	30	90,91	1,000
1 metastatic node	5	5,95	3	5,88	2	6,06	
2 metastatic nodes	3	3,57	2	3,92	1	3,03	
3 metastatic nodes	1	1,19	1	1,96	0	0,00	
HER-2 (n=113)							
Negative	84	74,34	54	83,08	30	62,50	0,023^*^
Indeterminate 2+	2	1,77	1	1,54	1	2,08	
Positive	27	23,89	10	15,38	17	35,42	
Immunohistochemical like subtypes (n=78)							
Luminal A	32	41,03	25	47,17	7	28,00	0,040^*^
Luminal B	25	32,05	18	33,96	7	28,00	
Luminal Her	7	8,97	5	9,43	2	8,00	
Pure Her	6	7,69	1	1,89	5	20,00	
Triple negative	8	10,26	4	7,55	4	16,00	
Hormonal receptors positive (>10) Her negative (n=78)							
No	21	26,92	10	18,87	11	44,00	0,029^*^
Yes	57	73,08	43	81,13	14	56,00	
Type of surgery (n=115)							
Lumpectomy	89	77,39	55	84,62	34	68,00	0,044^*^
Mastectomy	26	22,61	10	15,38	16	32,00	

VAB, vacuum assisted biopsy; OVAB, ordinary vacuum assisted biopsy; EVAB, enlarged vacuum assisted biopsy; n, absolute frequency; %, percentage; F, Fisher’s Exact Test; * statistical significance (p ≤ 0.05). MMG, mammography.

Bold values: Statistically significant.

When comparing EVAB and OVAB groups, significant differences were identified in several variables. The majority of EVAB procedures were ultrasound-guided, whereas most OVAB procedures were stereotactically guided. Masses were more frequently sampled with EVAB, while calcifications predominated in OVAB cases. Pathological findings of IC + DCIS were more common in EVAB, whereas DCIS was predominant in OVAB. Also, in final pathology IC + DCIS were more common in EVAB, whereas DCIS was predominant in OVAB. Although lumpectomy was the most frequent surgery in both groups, EVAB cases had a statistically higher lumpectomy rate than OVAB (**Table 3**).

### 3.2 Diagnostic test performance

The comparison between VAB LR-DCIS and surgical gold standard pathology is shown in **Table 4**. VAB LR-DCIS upstaging rate was 33.33%.

**Table 4:** VAB LR-DCIS comparison to surgical pathology.

VAB IC/DCIS	VAB LR-DCIS	Surgery LR-DCIS	Overall upstaging	HR-DCIS upstaging	IC upstaging
116(100%)	15(12.9%)	10(8.6%)	5(4.3%)	3(2.6%)	2(1.7%)

VAB, Vacuum assisted biopsy.

The calculated sensitivity, specificity, positive predictive value (PPV), negative predictive value (NPV) and accuracy of VAB, OVAB and EVAB, compared with the surgical gold standard are shown in **Tables 5** and **6**.

**Table 5:** Contingency table comparing results from VAB, OVAB, EVAB vs. surgery (Gold Standard).

Diagnostic Test		Final diagnosis after VAB and surgery		
		HR-DCIS/IC	LR-CDIS	Total
VAB	HR-DCIS/IC	101	0	**101**
	LR-DCIS	5	10	**15**
	**Total**	**106**	**10**	**116**

		Final diagnosis after ordinary VAB and surgery		
		HR-DCIS/IC	LR-DCIS	Total
OVAB	HR-DCIS/IC	43	0	**43**
	LR-CDIS	2	6	**8**
	**Total**	**45**	**6**	**51**

		Final diagnosis after EVAB and surgery		
		HR-DCIS/IC	LR-DCIS	Total
EVAB	HR-DCIS/IC	58	0	**58**
	LR-CDIS	3	4	**7**
	**Total**	**61**	**4**	**65**

VAB, vacuum assisted biopsy; OVAB, ordinary vacuum assisted biopsy; EVAB, enlarged vacuum assisted biopsy.

Bold values: Statistically significant.

**Table 6:** VAB, OVAB, EVAB results.

	VAB (IC 95%)	OVAB (IC 95%)	EVAB (IC 95%)
Sensitivity	95.3% (89.3–98.5)	95.6% (84.9–99.5)	95.1% (86.3–99.0)
Specificity	100% (69.2–100)	100% (54.1–100)	100% (39.8–100)
PPV	100% (96.4–100)	100% (91.8–100)	100% (93.8–100)
NPV	66.7% (38.4–88.2)	75.0% (34.9–96.8)	57.1% (18.4–90.1)
Accuracy	95.7% (90.2–98.6)	96.1% (86.5–99.5)	95.4% (87.1–99.0)

VAB, vacuum-assisted biopsy; OVAB, ordinary VAB; EVAB, enlarged VAB; PPV, positive predictive value; NPV, negative predictive value; IC95%, 95% confidence interval.

The 15 VAB LR-DCIS cases matched surgical outcome and COMET criteria are outlined in **Table 7**.

**Table 7:** VAB LR-DCIS cases matched surgical outcome and COMET criteria.

VAB FN LR-DCIS pathology	Age (years)	Image finding	VAB	Surgery	Surgical pathology	Staging (21)	COMET criteria (18)
1- DCIS (NG2)	58	Calcifications	OVAB	Lumpectomy	DCIS (NG3; HR-; HER 2-)	pTis(13mm)	Inclusion
2- DCIS (NG2)	38	Calcifications	EVAB	Mastectomy + SNB	DCIS (NG3; HR+; HER 2-)	pTis(20mm) pN0	Exclusion
3- DCIS (NG2)	59	Calcifications	EVAB	Lumpectomy + SNB	IDC (HG3; HR+; HER 2-; Ki 67 55%)	pT1b(6mm) pN0sn	Inclusion
4- DCIS (NG1)	57	Mass	EVAB	Lumpectomy	DCIS (NG3; HR+; HER 2-)	pTis(25mm)	Exclusion
5- DCIS (NG2)	66	Calcifications	OVAB	Lumpectomy	IDC (HG2; HR+; HER 2-; Ki 67 5%)	pT1(2mm) N0.	Inclusion
VAB TN LR-DCIS pathology							
1- DCIS (NG1)	46	Calcifications	EVAB	Lumpectomy	DCIS (NG1; HR+; HER 2-)	pTis(2,5mm)	Inclusion
2- DCIS (NG2; HR+; HER 2-)	31	Mass	EVAB	Lumpectomy	No residual tumor	pTis(4mm)	Exclusion
3- DCIS (NG1; HR+; HER 2-)	70	Calcifications	OVAB	Lumpectomy	No residual tumor	pTis(3mm)	Inclusion
4- DCIS (NG2; HR+; HER 2-)	53	Mass	EVAB	Lumpectomy	No residual tumor	pTis(4mm)	Exclusion
5- DCIS (NG2)	70	Calcifications	OVAB	Lumpectomy	DCIS (NG2; HR+; HER2-)	pTis(5mm)	Inclusion
6- DCIS (NG2; HR-; HER 2+)	62	Calcifications	OVAB	Lumpectomy	DCIS (NG2; HR-; HER 2+)	pTis(2mm)	Exclusion
7- DCIS (NG2; HR-; HER2-)	60	Calcifications	OVAB	Lumpectomy	No residual tumor	pTis(4mm)	Exclusion
8- DCSI (NG1; HR+; HER-)	45	Calcifications	OVAB	Lumpectomy	No residual tumor	pTis(2mm)	Inclusion
9- DCIS (NG2)	46	Calcifications	OVAB	Mastectomy + BLS	DCIS (NG2; HR+; HER 2-)	pTis(14mm) pN0sn	Inclusion
10- DCIS (NG2)	62	Mass associated to calcifications	EVAB	Lumpectomy	DCIS (NG2; HR+; HER 2-)	pTis(8mm)	Exclusion

FN, false negative; TN, true negative; VAB, vacuum assisted biopsy; OVAB, ordinary vacuum assisted biopsy; EVAB, enlarged vacuum assisted biopsy; DCIS: ductal carcinoma *in situ*; IDC, invasive ductal carcinoma; HR-DCIS, ductal carcinoma *in situ* with high risk of progression to invasive cancer; LR-DCIS, ductal carcinoma *in situ* with low risk of progression to invasive cancer; HR, hormone receptor; NG, nuclear grade; HG, histological grade; SNB, sentinel node biopsy.

There were 5 false-negative (FN) LR-DCIS cases identified across VAB. Of these, 3 cases (60%) occurred in EVAB, and 2 cases (40%) occurred in OVAB. Among the FN cases, 3 (60%) were upgraded to HR-DCIS, and 2 (40%) were upgraded to IC. The majority (80%) of FN cases were in patients over 40 years old, with 1 case (20%) in a patient under 40 years. Imaging findings included grouped calcifications in 4 cases (80%) and a mass in 1 case (20%). All lesions were ≤25 mm in size. Surgical management included 4 lumpectomies (80%) and 1 mastectomy (20%), with sentinel node biopsy (SNB) performed in 2 cases (40%) and no axillary evaluation in 3 cases (60%) (**Table 7**).

Of the 3 patients upgraded to HR-DCIS, 1 had ER/PR/HER2-negative status, 1 was under 40 years old, and 1 presented with a mass on imaging. Among the 2 cases upgraded to IC, one was an invasive ductal carcinoma (IDC) pT1b (6 mm, HG3, ER 100%, PR 5%, HER2-negative, Ki67 55%) pN0sn, and the other was pT1a (IDC, 2 mm, HG2, ER 100%, PR 100%, HER2-negative, Ki67 5%) N0 (**Table 7**).

There were 10 true-negative (TN) cases of LR-DCIS. Of these, 9 cases (90%) involved patients over 40 years of age, and 1 case (10%) involved a patient under 40. Imaging findings included calcifications in 7 cases (70%), masses in 2 cases (20%), and a mass associated with calcifications in 1 case (10%). Procedural distribution revealed that 6 cases (60%) were diagnosed using OVAB, and 4 cases (40%) were diagnosed using EVAB. Surgical management included 9 lumpectomies (90%) and 1 mastectomy (10%). SNB was performed in 1 case (10%), with no axillary procedure in the remaining 9 cases (90%). Complete resection of DCIS during biopsy was achieved in 5 cases (50%), comprising 3 OVAB cases and 2 EVAB cases.

When the whole cohort is compared with the COMET trial criteria, 7 cases (46.7%) would have been excluded: 2 cases (13.3%) due to age under 40, 4 cases (26.6%) due to mass findings on imaging, and 2 cases (13.3%) due to hormone receptor status (1 triple-negative and 1 HR+, HER2+). Of notice: 1 of these cases was a 31-year-old patient presenting with a mass (18) (**Table 7**).

**Figure 2:**
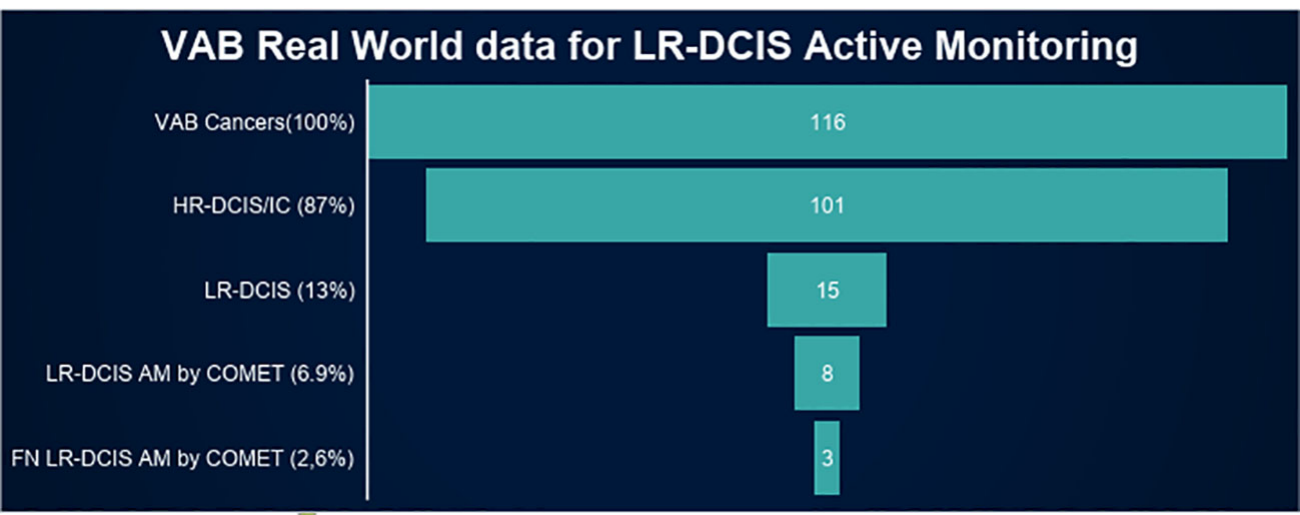
VAB real world data for LR-DCIS active monitoring. VAB, Vacuum assisted biopsy; HR-DCIS, Ductal carcinoma *in situ* with high risk of progression; LR-DCIS, Ductal carcinoma *in situ* with low risk of progression; AM, Active monitoring.

VAB LR-DCIS AM, according to COMET, would represent 6.9% (8/116) of all VAB cancers with 2.6% (3/116) underdiagnosed cases: 2 pT1a-bN0 hormone receptor positive breast cancers and 1 HR-DCIS (**Figure 2**).

## 4 Discussion

Our series, as far as we know, is the first to evaluate the impact of EVAB on the accurate diagnosis of LR-DCIS for active monitoring. Although extended vacuum procedures, such as EVAB and VAE, reduce the upgrade rate of DCIS diagnosed with biopsy to invasive cancers in surgery when compared with CNB (30), EVAB did not reduce upgrade rate of LR-DCIS to HR-DCIS or IC in surgery compared with OVAB in our series. EVAB was more frequent in masses and therefore was mostly guided by US. It is well known that presence of a mass increases the risk of VAB DCIS upstaging to IC in surgery (32). The lack of statistical difference between OVAB and EVAB could be explained by some potential selection bias. On the other hand, the results strengthen the recommendation of mass as exclusion criteria for VAB LR-DCIS AM (18).

Our series demonstrated that, in real-world practice based on conventional eosin-hematoxylin pathology, the upstaging of VAB for HR-DCIS/IC was as high as 33.33%. Moreover, there was no significant improvement in upstaging with the extension of the vacuum procedure: EVAB (42.85%) versus OVAB (25%). Demographic, epidemiological, clinical, imaging and immunohistochemistry selection criteria are critical for improving VAB’s diagnostic accuracy and reducing false negative rate (FNR). In our study, the NPV of VAB for HR-DCIS/IC was 66.7%, a value directly influenced by the high prevalence of HR-DCIS/IC in the sample. Our upstaging rate was higher than reported in previous studies (6, 8, 10, 12), although those studies did not specifically evaluate NPV. Although VAB demonstrated excellent sensitivity and specificity, the probability that a negative result truly indicates the absence of HR-DCIS/IC remains limited. Furthermore, we highlight that in high-prevalence populations, a negative VAB result should be interpreted with caution and always considered in conjunction with rigorous active monitoring strategies.

Several trials have evaluated AM for LR-DCIS, each employing distinct inclusion and exclusion criteria (6–10, 12, 16, 18). Regarding diagnostic procedures, while VAB is included as an acceptable diagnostic modality in COMET (7, 18), LORIS (6, 8), and LORD (16) trials, none distinguish between OVAB, EVAB, or vacuum-assisted excision (VAE). The COMET trial allows inclusion of LR-DCIS diagnosed via CNB or VAB without restrictions on the number of samples (7, 18). LORIS mandates at least a 12G needle for VAB, again with no restrictions on the number of samples (6, 8). LORD is unique in requiring a minimum of 6 samples with an 8-9G needle or 12 samples with a 10-11G needle (16).

Retrospectively VAB LR-DCIS upstaging risk to HR-DCIS/IC in surgery varies from 5% to 12% according to inclusion criteria, LORD, LORIS, COMET (6, 8, 10). These trials apply different inclusion criteria beyond just conventional HE pathology. In our series, VAB LR-DCIS upstaging to HR-DCIS/IC was high (33.33%) probably because it considered just conventional, HE pathology. So, it is very important to associate clinical, imaging and immunohistochemistry data to refine the selection criteria of VAB LR-DCIS AM.

Despite the retrospective data, COMET prospective published data demonstrated that two years incidence of IC was 8.7% in the LR-DCIS guideline-concordant care (surgery with or without radiation therapy) versus 3.1% in the AM group, leading the inference that IC upstaging would be approximately 8.7% in the AM group (18). In our series, applying COMET criteria, VAB LR-DCIS upstaging rate to IC was 28.6, higher than COMET. The limited number of the sample could explain the difference.

Of the 15 cases of LR-DCIS identified on VAB, 5 were completely excised by the biopsy. Complete pathological excision of LR-DCIS during biopsy eliminates the possibility of an upgrade during surgery and ensures the safety of AM. In this context, vacuum assisted excision (VAE) may enhance the oncological safety of active surveillance by reducing the underestimation inherent in percutaneous needle diagnosis and represents an approach warranting consideration in future trials (33).

Based on COMET inclusion criteria (18), 2 (40%) upgraded false-negative (FN) cases from our series would be excluded, leaving only 3 (60%). Of these, one was classified as pT1a (IDC, 2 mm, G2, ER 100%, PR 100%, HER2-negative, Ki-67 5%), one as pT1b (IDC, 6 mm, G3, ER 100%, PR 5%, HER2-negative, Ki-67 55%) pN0sn and one as HR-DCIS. Small, luminal stage I cancers (pT1a-bpN0) were the typical upgraded invasive malignancies observed in prior series (6, 8) and COMET trial (18). In COMET 94.7% of invasive cancers that were diagnosed in 2 years of AM were ER positive and 52,6% <1.1cm. For these patients, sentinel lymph node biopsy (SNB) can be safely omitted (34–36), and hormone therapy or radiation therapy, alone or combined, may suffice for disease control. In accordance, our series demonstrates the reproducibility of COMET in real world practice.

The upstaging of VAB LR-DCIS in our series was 33.33%, higher than reported in other studies (6, 8, 10, 12). Literature indicates significant interobserver variation in the classification of LR-DCIS (37–41). Besides, there is always the chance of misdiagnoses in VAB. Our results highlight the potential need for a double reading of pathology reports prior to initiating LR-DCIS AM. The COMET trial required concordance between two clinical pathologists to mitigate interobserver variation (7, 18). Another potential strategy to address interobserver variation and reduce FNR is the use of artificial intelligence (AI), which is currently under evaluation and development (42).

Demographic, epidemiological, clinical, imaging and immunohistochemistry selection criteria are critical for improving VAB’s diagnostic accuracy and reducing FNR. When COMET trial criteria were applied to our series, 7 patients (6.0%) would be excluded, leaving 8 (6.9%) eligible for AM, of whom 3 (2.6%) would represent FN cases of LR-DCIS. Thus, in real-world practice from April 13, 2017, to November 28, 2020, AM for VAB LR-DCIS applying COMET criteria would reduce approximately 8 (6.9%) cases of breast cancer overtreatment, counterbalanced by 3 (2.6%) potentially undertreated HR-DCIS/IC. Consequently, 7 patients (6.0%) would have avoided lumpectomy, 1 (0.8%) mastectomy and 2 (1.7%) sentinel node biopsy.

Our study has some limitations. In our study, the NPV represents the probability that a lesion diagnosed as LR-DCIS by VAB truly does not correspond to HR-DCIS or IC at final surgery. It is important to note that the 95% confidence intervals for the NPV were wide (e.g., 18.4–90.1% for EVAB), reflecting both the high prevalence of HR-DCIS/IC and the relatively small number of truly negative cases. This finding underscores the need for cautious interpretation of negative VAB results in the present study, particularly in the context of AM strategies.

Of the 133 VAB cancers found, 17 were excluded due to lack of surgical pathology report. Although it could lead to selection bias, it is quite improbable. As the whole cohort, these were cases of IC/DCIS and the prevalence of VAB LR-DCIS was 12.9%. It was expected to be around 2 more cases of VAB LR-DCIS. The upstage rate would range from 29.4% (2 TN) to 41.2% (2 FN), still high and comparable to the 33.33% found. COMET allowed inclusion of patients diagnosed with LR-DCIS by CNB, VAB and diagnostic open surgery. Our series is restricted to VAB. Although the limited size of the sample, the findings are still valuable and reflect real world practice. The analysis was retrospective and there were differences between OVAB and EVAB cohort that could lead to potential selection bias. A prospective trial to evaluate OVAB versus EVAB or even VAE for LR-DCIS diagnosis would be recommended.

## 5 Conclusion

VAB LR-DCIS active monitoring based on COMET criteria would lead to a moderate overall reduction (6.9%) of short-term breast cancer surgical overtreatment counterbalanced by a low rate (2.6%) of underdiagnosed HR-DCIS/IC potentially treatable by adjuvant hormone therapy in real world clinical practice. The diagnosis of LR-DCIS using VAB, based on conventional pathology, demonstrates a low negative predictive value (NPV) for high-risk DCIS (HR-DCIS) or invasive carcinoma (IC) in real-world clinical practice. EVAB is not superior to ordinary VAB in reducing the underdiagnosis of HR-DCIS/IC.

## Data Availability

The original contributions presented in the study are included in the article/supplementary material. Further inquiries can be directed to the corresponding author.

## Glossary

DCIS: ductal carcinoma in situ
LR-DCIS: ductal carcinoma *in situ* with low risk of progression to invasive cancer
IC: invasive cancer
VAB: vacuum assisted biopsy
HR-DCIS: ductal carcinoma *in situ* with high risk of progression to invasive cancer
PPV: positive predictive value
NPV: negative predictive value
FNA: fine needle aspiration
CNB: core needle biopsy
BI-RADS: Breast Image Reporting and Data System
B3 lesions: lesions with indeterminate potential of malignance in core needle biopsy according to the Royal College of Pathologist
TI: maximum imaging tumor size
HE: hematoxylin-eosin
FISH: Fluorescence *In Situ* Hybridization
ER: estrogen receptor
PR: progesterone receptor
HER 2: Human epidermal growth factor receptor 2
Ki-67: protein Ki-67
SNB: sentinel node biopsy
COMET: Comparing an Operation to Monitoring, with or without Endocrine Therapy for low-risk ductal carcinoma *in-situ* (DCIS) of the breast
T: pathological tumor size
US: ultrasound
MMG: mammography
FN: false negative
TN: true negative
VAE: vacuum assisted excision
VAE Breast 01: Vacuum Assisted Excision, A single step approach to the diagnosis and treatment of lesions of indeterminate potential of malignance and early breast cancer
CMSH: Cavity margin sample shaving
AI: Artificial intelligence.
